# Supplementary material for: Diversity of Salmonella enterica isolates from urban river and sewage water in Blantyre, Malawi
Source: PLoS Negl Trop Dis. 2025 Sep 9;19(9):e0012413. doi: 10.1371/journal.pntd.0012413 (PMC12431665; doi:10.1371/journal.pntd.0012413)
Supplement: Attachment — Submitted filename: Salmoella Manuscript Review.docx [file pntd.0012413.s010.docx]

| Manuscript number | PNTD-D-24-01094 |
| --- | --- |
| Full title | Diversity of Salmonella enterica isolates from urban river and sewage water in  Blantyre, Malawi. |
| Short title | Diversity of Salmonella enterica from environmental samples in Malawi. |
| Article Type | Research Article |
| Reviewer |  |
| Summary |  |
| The paper analyses samples collected from the environment, in urban river (87.9%) and sewage water (8.85%) in Blantyre Malawi. The initial surveillance was for *Salmonella* Typhi from 2019-2021. However further investigation for *Salmonella* Typhimurium was done on the samples based on which this analysis was done. A total of 1,042 samples of NTS were identified of which 339 were sequenced of which 270 genomes passed quality check. The paper demonstrated that S. Typhi ca be cultured from water river samples.  The aim of the paper was to determine the diversity of NTS isolates from urban environments. | |
| Comments |  |
| The short title | The short title suggest that the research is being done on the samples rather than on the environment |
| 96 | ‘Typhi from natural river water’ should either include all of the environmental sources or just say from the environment |
| 101 | 1. The paper has not defined the ‘diversity’ being studies as such it is difficult to measure whether the objectives have been achieved or not. 2. Was there any differences in the diversity among difference environmental sources, if yes what were they and what can we learn from it? |
| Methods | DNA extraction and genome sequencing techniques are standard. However, because the objectives of the paper have not been spelt out, the bioinformatics analysis of the methods is not clear what it is addressing. It is difficult to map the methodology to aim and critique it whether it is the right approach to address the objectives to give the desired result. |
| Results | Because the objectives of the study were not clearly defined, it is difficult to conclude where satisfactory results have been found. Table 2 is best suited in the appendix due to the size which spans 3 pages unless, it is edited to fit on one page. |
| 308 | The claim that this is the first time typhimurium ST313 is identified in rivers needs to be substantiated. There is need for evidence of previous studies which failed to find ST 313. |
| 316 | 1. While the authors claim that evidence of ST313 in the rivers suggest transmission and infections in humans and animals they have not laboured to show the link with strains from patients at the hospital. 2. Is there evidence of human activity in these water streams and sewage to justify transmission or could these findings suggest shedding? |
| 325 | Can the authors show evidence of S. Isangi among patients to show the link between the environment and transmission to humans? |
| 332 | Authors needs to explain why some serovars found in the environment are not found in patients in Malawi. |
| 350 | How do these limitations affect the results? Can authors suggest how they could have done the same study different to mitigate the limitation. |
| Figures | The quality of the figures is very poor and needs improvement |
