# Supplementary material for: Diversity of Salmonella enterica isolates from urban river and sewage water in Blantyre, Malawi
Source: PLoS Negl Trop Dis. 2025 Sep 9;19(9):e0012413. doi: 10.1371/journal.pntd.0012413 (PMC12431665; doi:10.1371/journal.pntd.0012413)
Supplement: Attachment — Submitted filename: PNTD-D-24-01094 Response to Reviewers.docx [file pntd.0012413.s011.docx]

PNTD-D-24-01094 Response to Reviewers

Dear Grace, Ana, Shaden and Paul,

Thank you for the response. As requested, I have uploaded two copies of the revised document, one with and one without tracked changes. Please see the comments and our responses to each of the reviewer’s comments below. All line references in responses are done to “Simple Markup” or the clean version of the document, whilst comments retain their original line numbers as per the received responses.

**Editor comments**

1. CRediT Author Contributions: These have been added to the submission form as requested.
2. As requested, TM has been removed from page 10, previously, page 7.
3. We confirm all information that has been presented in this work has been uploaded to the database linked, which relates each isolate to their Accession number from EDI, so that all sequences can also be found. In addition, the statement “All available data has been uploaded”, we included a data repository link and DOI rather than a large data set attached to the end of the document. This statement can be found on page 24.
4. As requested, the grant code that was listed in the document and on the submission, form have been correct to be identical. The only author to receive funding in relation to this study is senior author, Professor Nicholas Feasey.

**Reviewer #1 comments**

1. Please detail how sites were selected for environmental surveillance as well as the timelines for surveillance of different sites. A map may be helpful here.

The relevant articles have been cited, along with a map added in Supplementary Materials. Adding details to how the GIS was done for site selection would increase the length of the paper significantly. A summary has been added on Lines 120 to 131, including a new figure of the sample sites mapped and read as:

“The environmental surveillance study team collected 2,693 samples of water, Moore swabs, soil and biofilms over an eighteen-month period between June 2019 to January 2021. Sites were selected initially based on water usage points close to predicted typhoid clusters based on geolocation data from patients admitted to Queen Elizabeth Central Hospital with culture confirmed typhoid fever using the analysis from Gauld *et al.,* 2022 [21, 22] plus the inclusion of a wastewater treatment plant. A map of Blantyre with the sampling locations can be found in Figure 1.

**Figure 1:** Locations of all sampling sites used in Blantyre, Malawi, during water surveillance between 2018 to 2020, depicting both natural water (●) and wastewater (◆) sites. Figure created in QGIS using Carto Voyager basemap CC BY-NC-ND 4.0 (https://tiles-a.basemaps.cartocdn.com/#12/-15.7845/35.0179) and Map data from OpenStreetMap CC BY-SA 2.0 (https://www.openstreetmap.org/#map=14/-15.79604/35.00596). The Malawi district shape file from GADM CC-BY (https://gadm.org/index.html). Sampling Locations described in Gauld *et al.,* 2022 [21, 22] and Rigby *et al*., 2022 [18].”

2. Line 39: The statement “These environmental iNTS isolates did not, however, cluster with pathovariants contemporaneously associated with bloodstream infection “ appears to contradict statements made in lines 221-236. Please consider rephrasing to clarify the intended meaning.

The statement highlighted by the reviewer has been removed from the abstract.

3. Lines 53 & 57: Please capitalise and italicise Salmonella

This has been acknowledged and completed. For reference, they are now lines 52 and 56

4. Line 99: A space appears to be missing between “on” and “Salmonella”

This has also been corrected, and is now line 100

5. Line 111: I think a word may be missing here

This missing word has been added in on like 133: “…published **in** Rigby *et al*., 2022”

6. Line 115: Please include a supplementary table of PCR primers used, or a reference to a publication that details these. I believe the primers may be those detailed in reference #16 but this is not clear at present. If they are indeed those from reference #16, it may be helpful to briefly reiterate key aspects of “pathway P” and other methods from reference #16 in the manuscript text so the reader does not have to look these up to understand the approach used

Due to other comments from reviewers, a much more in depth description of the real time PCR used is available in the text on lines 152 to 169. Additionally, a supplementary table (4) has been added with full details of the primer and probe sequences, the gene these target including gBlocks used for optimisation work, mastermix concentrations and thermocycling conditions.

“Isolates were screened using a real time PCR (qPCR) adapted from Nair *et al*., 2019 [17], adopting only the gene targets relevant for *S*. Typhi detection (Supplementary Table 4[18, 27, 28]). This assay used a triplex assay targeting *ttr*, a pan-salmonella gene, found in all subspecies and serovars of both species of *Salmonella*: *enterica* and *bongori*, involved in tetrathionate respiration (AF282268 [29]), *­tviB*, a *S*. Typhi specific component gene of the *viaB* locus, which is involved in the synthesis, transport and expression*­* of the Vi antigen (NC_003198 [17]) and *staG*, also an *S*. Typhi specific gene designed for use with blood culture for diagnostics*,* which is involved in fimbriae production (AL513382 [16]).

Isolates which were *ttr* positive were archived as NTS, whilst any isolates that contained *staG* or *tviB* were then further verified using a biochemistry test for Enterobacteriaceae, that could distinguish between NTS and Typhoidal *Salmonella* spp. (Biomerieux API 20E), and serology using the Kaufmann-White scheme, (O12, O9, Vi and Hd) to confirm whether they were *S*. Typhi, *S*. Typhimurium, *S*. Enteritis or other or NTS [24, 30-32].

A total of 1,048 *Salmonella enterica* isolates were obtained from 425 culture positive samples, including six *S*. Typhi, identified by the qPCR, biochemistry and serology detailed above. The remaining 1,042 isolates were confirmed to be NTS using previously published PCR primers; specifically isolates that were PCR positive for the pan-*Salmonella* marker *ttr* alone or with either (but not both) *tviB* and *staG* additionally being PCR positive (Table 1).”

7. Line 144: Please consider detailing any QC cutoffs used here

Thank you for this comment. Please find the cut-offs for quality control detailed on lines 218-221

8. Line 153 & 159 & 162: Please detail how the QC thresholds used were selected

Our samples were prepared in the laboratory in Malawi before shipping to the UK for sequencing. Initial kraken2 and CheckM screening of the data indicated there was some degree of contamination in the samples submitted for sequencing. To mitigate this, we applied filters (>70% of sequencing reads must be *Salmonella* using Kraken, <20% contamination using CheckM on the assemblies. This is more permissive than is typical but reflects a balance between retaining samples and minimising the impact of any potential contamination. We further mitigated contamination by using a stringent core gene alignment approach with Roary, where we required Core genes to be present in 100% of genomes. This would be expected to minimise the impact of contamination in the core gene alignment we used for the phylogeny and species classifications.

There is previously published data which has been analysed using these parameters:

- CheckM (<90% completeness and >20% contamination) has been previously published by Parks (<https://pmc.ncbi.nlm.nih.gov/articles/PMC4484387/>).
- Kraken, genomes with <70% abundance of *Salmonella* were excluded from further analysis, as previously published by Perez-Sepulveda (<https://genomebiology.biomedcentral.com/articles/10.1186/s13059-021-02536-3>).
- QUAST; Enterobase (<https://journals.plos.org/plosgenetics/article?id=10.1371/journal.pgen.1007261>)
- Perez-Sepulveda (<https://genomebiology.biomedcentral.com/articles/10.1186/s13059-021-02536-3>). These citations have been added into the text on lines 284, 289 and 291.

9. Line 157: Please provide a citation for the WSI pipeline or details of the methods/software used. Please also detail which assemblies from which tools were used in the final analysis. If only assemblies from SPAdes were used, then perhaps the remaining methods details can be removed?

Thank you for the comment. The citation for the WSI pipeline, which has been added to the relevant text on page 11, is;

Page, A. J. et al. Robust high-throughput prokaryote de novo assembly and improvement pipeline for Illumina data. Microb. Genom. 2, e000083 (2016).

The software used to assemble the data was SPAdes v3.15.5 and PROKKA v1.14.5 was used to annotate the genomes, as detailed within the text, therefore we have retained the relevant information within the text.

10. Line 164: Please include a citation for SISTR

This has been included on line 222, The citation for SISTR is:

The Salmonella In Silico Typing Resource (SISTR): an open web-accessible tool for rapidly typing and subtyping draft Salmonella genome assemblies. Catherine Yoshida, Peter Kruczkiewicz, Chad R. Laing, Erika J. Lingohr, Victor P.J. Gannon, John H.E. Nash, Eduardo N. Taboada. PLoS ONE 11(1): e0147101. doi: 10.1371/journal.pone.0147101

11. Line 167: I was unable to find details of the cutoffs used in Table S1 – this instead appears to be a list of distinct isolates?

This was an editing issue from Co-Author comments, the line identified by the reviewer has been removed.

12. Line 172: I think this sentence may need to start with “A” and “were” should be “was”?

This section is now in Line 233, Completed as requested.

13. Line 175: Please detail the models used for tree inference e.g. GTR gamma etc.

We used RAxML v8.2.8 with the GAMMA model and 100 bootstrap replicates. We have now added this information to the manuscript on Lines 234 to 238:

“RAxML (Randomised Accelerated Maximum Likelihood) (version 8.2.8 [46]) was run on the resulting core SNP-alignment to construct a maximum likelihood tree using the core gene SNP alignment of all isolates passing QA . Reliability of inferred branch partitions was assessed with 100 bootstrap replicates using the Infinitely Many Genes model as part of Panaroo [47, 48]. The tree was visualised using ggtree [49] (version 3.2).”

14. Lines 177-183: It’s unclear from the current text why the “duplicate” samples were pruned from the tree – was this for ease of visualisation or it to have “representative” genomes from each sample? Please clarify the rationale in the manuscript text

This is now discussed in lines 239 to 245, with some further information earlier at 116 to 119. In brief, duplicates were isolates that were both from the same sample and were genomically identical, and therefore not representative of abundance due to multiple culture steps involved.

“During isolation, any sample that yielded multiple colonies with *Salmonella* spp. morphology had up to 10 colony “picks” sub-cultured due to the low abundance of *S*. Typhi within positive samples, and number of competing salmonellae in each sample, therefore, it was assumed that if the two isolates were genomically identical and from the same sample, then they were subcultures of the same organism. A ‘duplicate’ was defined as two genomes, collected from the same sample, which were between 0-2 pairwise SNP distance within the core gene alignment calculated using snp-dists (<https://github.com/tseemann/snp-dists>) [50], and therefore consequently also the same serovar and ST type. ‘Duplicated’ genomes were removed from the tree.”

15. Line 188-189: It is unclear why long reads are mentioned here, presumably the analysis was carried out using short reads only? If hybrid assemblies were used then details of these need to be included at the appropriate section.

Our apologies, this was an error. We used publicly available closed reference genomes for mapping analysis within *S. Typhimurium ST313 and S. Enteritidis ST11*. There were no long read or hybrid assemblies.

16. Line 192: Please include details of how recombination filtering was conducted e.g. using Gubbins (PubMedID/PMID: 25414349) or similar here

For species level analysis, we used a core gene alignment approach (Panaroo [47]) which partially mitigates the impact of recombination. Tools such as Gubbins are specifically not recommended for use with core gene alignments because they rely on positional synteny which is lost during the core gene alignment process. Moreover, we were not inferring finescale within-lineage relationships where recombination would be important (note that tools such as Gubbins are only designed for within-lineage analysis), but high-level clustering across *Salmonella enterica* - at this evolutionary scale the role of recombination is likely indistinguishable from that of vertical inheritance (Sakoparnig et al 2021, <https://elifesciences.org/articles/65366>), and has no bearing on the broad species level relationships described in the main tree.

17. Line 193-4: Please detail the specific outgroups used here – I realise some of this detail is captured in supplementary figure legends, but it would be helpful to mention here

Thank you for this comment. This information has been kept in the legend of the supplementary material to avoid duplication.

18. Line 198: Please include a table detailing the contextual genomes used in the study.

Apologies that this was not included in the original submission. Two tables of contextual genomes, one of *S*. Typhimurium ST313 and a second of *S*. Enteritidis ST11 have been attached to this resubmission as Supplementary Tables 5 and 6 respectively.

19. I also think “measurement” may need to be “measurements” here

Changed as suggested on Line 266

20. Line 209-211: I could only identify n=15 mixed samples detailed in table S2 - perhaps an extra column could be added to highlight this information more clearly? I also got the same result looking over table 2. Moreover, please note that these mixed samples should have been excluded from phylogenomic analysis, and it would be helpful to clarify this in the manuscript text

Thank you for identifying this issue, I had counted the headers of the table as an additional sample and corrected this accordingly.

21. Figure 1: This is a nice figure but please note that many of the colours used here are very similar so it is difficult to discern some serovars and STs from others. Perhaps text labels could be used here instead or only the most common types shown with colours, with all other minor types coloured a single colour for “other”? The text is also quite blurred on my copy of this figure but this may be due to the compression used by the journal system.
    - Please also note that it may be possible to remove the *tviB* coloured bar from the heatmap as the gene appears absent in all samples shown.
    - Please also note that the scale bar label overlaps the tree – this may be readily fixed in illustrator if the file is exported from R as a pdf, but can also be fixed in ggtree code.

Thank you for your comment. Due to the extraordinary diversity of the *Salmonella* serovars which were detected, aside from a variety of colours, it is challenging to display the serovars and STs in such a way that allows us to demonstrate this diversity within the figure. We have now added clade labels to indicate the major serotypes and STs. To accommodate the additional space required, we now use tip-point colours to indicate subspecies. We have also moved the position of the scalebars to avoid overlap with the plots.

22. Line 232: Please consider providing a brief explanation of the “outlier clade” and a citation here so that readers do not have to follow up with this in other studies.

Thank you for this comment. The ‘outlier cluster’ described here is a reference to that first described by Feasey et al, a reference for which has been provided within the text (Reference number 5). As described within the paper of Feasey *et al.,* the outlier cluster refers to a clade which was not found to be associated with epidemics of clinical disease, but from which the Global Epidemic Clade emerged.

23. Line 233: Please consider providing a definition of MDR in the methods section – i.e. does this refer to co-resistance to a particular combination of drugs, or resistance to a number of different drug classes? I can see this is explained at line 255 onwards, but earlier mention at first use would help prevent confusion

This has been addressed earlier in the text as requested, and the description can be found at line 293.

24. Line 313: Please include comma(s) if using “however” mid-sentence

This change has been made, now at line 396.

25. Line 617: Please provide the accession number of the Gallarium genome used to root the tree

The accession number of the *S*. Gallinarum genome used to root the tree is SAMN08796416. This has been added to line 831.

26. Line 329 and elsewhere: Please review the use of capitalisation of salmonellae throughout for consistency.

All have been checked and changed where appropriate to lowercase located on Lines 70; 75; 95; 105; 189; 241; 282; 325; 412; 443 and 802. The one in line 70 is the start of a sentence and has remained capitalised as it is the beginning of a sentence, all others corrected to lowercase.

27. Line 308-315: At a SNP distance level, how closely related were the environmental NTS to those causing human infections? Can these data answer questions related to transmission dynamics raised in the discussion?

Thank you very much indeed for this comment. This is a very interesting question, but unfortunately falls outside of the scope of this paper. Additionally, it is not possible to use the data presented here to infer the dynamics of transmission of these particular samples; for this we would require a more intense sampling protocol and higher resolution genomic analysis. Instead, here we are able to observe that the genomic relationship between the environmental NTS sampled here to those causing clinical human infections is closer than to other comparable genomes. This would need further quantifying, such as by SNP analysis, as you suggest, and likely over a more intense sampling schedule, to be able infer whether or not direct transmission of these bacteria has occurred.

28. Supplementary AMR table: Please note that a trailing ‘Ê’ character in present in all accession numbers and some lane numbers. Also, it would be helpful to see ttr, staG, and tviB data for all genome data, not just those with AMR determinants. Perhaps these data could be added to the supplementary total genome IDs table? Regarding the AMR data shown – it would be better to code these as binary for presence/absence rather than S/R as the column headings are genes and not drugs.

Supplementary AMR table has been integrated into Total Genome ID’s table, renamed accordingly, and uploaded to the repository BioStudies: <https://www.ebi.ac.uk/biostudies/studies/S-BSST1695>. Data has been cleaned according to suggestions by all reviewers.

29. Supplementary total genome IDs table: Please also include individual sample accession numbers to make it easier for readers to link the information to the genomes in ENA/GenBank. Also, as the last four columns of this table are all NA, perhaps they are not needed?

As previously mentioned, the tables have been combined and cleaned up as suggested by reviewers.

**Reviewer #2 comments**

1. DNA extraction and genome sequencing techniques are standard. However, because the objectives of the paper have not been spelt out, the bioinformatics analysis of the methods is not clear what it is addressing. It is difficult to map the methodology to aim and critique it whether it is the right approach to address the objectives to give the desired result.

As per the reviewer’s request, we have emphasised the purpose of the paper at the end of the introduction, lines 102 to 108:

“The aim of this research was to use whole genome sequencing to determine the diversity of NTS isolated from water systems in Malawi with three specific objectives; firstly, to identify key human pathogenic serovars and multi-locus sequence types circulating in the environment, secondly, to establish which antimicrobial resistance determinants are present in salmonellae circulating in the environment. Thirdly, having isolated phenotypically confirmed NTS that gave positive amplification for gene targets previously proposed as being specific for *S*. Typhi, we aimed to identify which serovars were responsible for qPCR cross-reactivity. ”

2. Because the objectives of the study were not clearly defined, it is difficult to conclude where satisfactory results have been found. Table 2 is best suited in the appendix due to the size which spans 3 pages unless, it is edited to fit on one page.

As per Reviewer 2’s recommendation, we have moved Table 2 as it is currently presented into the supplementary materials. However, due to its importance in the results and discussion at this section of the paper, we have edited the table to a size that will fit on one page by removing any serovars that had less than three isolates associated with it.

3. The conclusion of the work is the supported by the data presented, however, there is need for clarity of the diversity being studied. This will help in determining if indeed the diversity being investigated has been established.

The aim of the original study was to identify *S*. Typhi from the environment, primarily in this case, from natural water sources in an endemic city, Blantyre, Malawi. The methods utilised yielded a high number of non-Typhoid *Salmonella*. The purpose of the data presented here is the determine what serovar, sequence type and antimicrobial resistance profiles are present amongst a subset of those NTS isolates and therefore inform us what NTS are co-circulating in the environment with *S*. Typhi (Rigby *et al*., 2022, Uzzel *et al*., 2023 and Uzzel *et al*., 2024).

4. The paper analyses samples collected from the environment, in urban river (87.9%) and sewage water (8.85%) in Blantyre Malawi. The initial surveillance was for Salmonella Typhi from 2019-2021. However further investigation for Salmonella Typhimurium was done on the samples based on which this analysis was done. A total of 1,042 samples of NTS were identified of which 339 were sequenced of which 270 genomes passed quality check. The paper demonstrated that S. Typhi can be cultured from water river samples.

This summary is accurate

5. I recommend: 'major revisions', before accepting the paper

We thank the reviewer for their insights and hope we have addressed their concerns.

**Reviewer #3 comments**

Methods

1. L111: how were the 6 Typhi identified in the 1048 salmonella isolates?

Lines 152 to 169 now details how these isolates were identified as *S*. Typhi prior to genome sequencing:

“Isolates were screened using a real time PCR (qPCR) adapted from Nair *et al*., 2019 [17], adopting only the gene targets relevant for *S*. Typhi detection (Supplementary Table 4[18, 27, 28]). This assay used a triplex assay targeting *ttr*, a pan-salmonella gene, found in all subspecies and serovars of both species of *Salmonella*: *enterica* and *bongori*, involved in tetrathionate respiration (AF282268 [29]), *­tviB*, a *S*. Typhi specific component gene of the *viaB* locus, which is involved in the synthesis, transport and expression*­* of the Vi antigen (NC_003198 [17]) and *staG*, also an *S*. Typhi specific gene designed for use with blood culture for diagnostics*,* which is involved in fimbriae production (AL513382 [16]).

Isolates which were *ttr* positive were archived as NTS, whilst any isolates that contained *staG* or *tviB* were then further verified using a biochemistry test for Enterobacteriaceae, that could distinguish between NTS and Typhoidal *Salmonella* spp. (Biomerieux API 20E), and serology using the Kaufmann-White scheme, (O12, O9, Vi and Hd) to confirm whether they were *S*. Typhi, *S*. Typhimurium, *S*. Enteritis or other or NTS [24, 30-32].

A total of 1,048 *Salmonella enterica* isolates were obtained from 425 culture positive samples, including six *S*. Typhi, identified by the qPCR, biochemistry and serology detailed above. The remaining 1,042 isolates were confirmed to be NTS using previously published PCR primers; specifically isolates that were PCR positive for the pan-*Salmonella* marker *ttr* alone or with either (but not both) *tviB* and *staG* additionally being PCR positive (Table 1).”

2. L113: reference needed for previously published PCR primers. It would also be useful to explain the significance of *ttr*, *staG* and *tviB* and why these are used as diagnostic markers for Salmonella

This is discussed on Lines 152 to 153, with further details of the qPCR reagents, including primer and gene sequences, in lines 153 to 159 and Supplementary Table 4.

3. The selection strategy for the 339 isolates for WGS should be revised to ensure clarity of numbers. All the S. Typhi. and then PCR positive for some genes of interest (n = 51?) and the n = 269 randomly selected ones. From L120-124 – correct in reading that these numbers from the total 339?

This has been rewritten for clarity and should now add up to the correct number of isolates submitted for sequencing, minus the six S. Typhi that were not analysed as a part of this study and is now discussed on Lines 170 to 181:

“Three-hundred-forty-one isolates were selected for whole genome sequencing (WGS). From the six samples that yielded isolates with positive amplification for all three primer targets we selected all 19 discreet single colonies. As the focus of this study is NTS, whilst these isolates had the gene targets for *S*. Typhi, this data set will only discuss those isolates that yield NTS from sequencing. Additional isolates of interest were chosen based on qPCR results, including all NTS isolates that were *ttr* positive plus either *staG* (n=23) or *tviB* (n=1) positive. A further 296 isolates were selected at random, attempting to ensure only one colony pick from each environmental sample chosen was submitted for WGS.

Of the 341 isolates selected for sequencing, 187 were grab samples, from rivers (n = 154) and wastewater (n = 33). Moore’s swabs accounted for 108 samples from rivers (n = 66) and wastewater (n = 42). The remainder 46 isolates were from biofilms and algae (n = 24), soil and sediment samples (n = 18) and food (n = 4).”

a. Please also define what is a ‘grab sample’.

Description for both composite and grab samples have been added in Lines 133 to 137:

“…samples collected were categorised as either composite samples, exposed to the water source over a longer period of time (Moore’s Swabs [25], soil/sediment samples and biofilms) and grab samples, snapshots of the water flowing through at the time of collection (one-litre water samples were collected from rivers, a wastewater plant, boreholes, water kiosks, and market produce washing buckets).”

b. Potentially a figure could be included to show the selection of isolates, potentially including a high-level map on the area.

We have added a map of the area, including all the sampling locations as a Figure 1. Too many locations are present to clearly show a breakdown of the location of each sample type, as during sampling, each type of sample was collected at each site. Not all samples per site had amplification on the qPCR, and the isolates, whilst distributed over time period and location, not all isolates were sent for sequencing.

c. Could be done instead of Table 1 as this table doesn’t include any of the information about water source.

Table 1 has now been updated to show the break down of sample type and qPCR result for all the samples selected for sequencing, found on Lines 182 to 183.

4. L154 – what reads were used that were published elsewhere? These aren’t in the supp tables provided and no citation provided.

Apologies that this was not included in the original submission. Two tables of contextual genomes, one of *S*. Typhimurium ST313 and a second of *S*. Enteritidis ST11 have been attached to this resubmission as supplementary tables 5 and 6.

5. L170-L171. What were the parameters for Roary? In particular the threshold for a ‘core gene’. Present in 99% of isolates? Or 95%? How many core genes were included for all the Salmonella isolates? Each of the core genes were first aligned (with mafft?) and the concatenated together?

Thank you for this comment. The pangenome inferred by Roary identified 16,677 gene orthologs, and we required a stringent threshold of 100% presence to be considered core (we had some concerns about potential contamination). Using this definition, there were 2,882 core genes used to construct the core gene alignment. The following sentence has been added in (Line 233) ‘A gene was considered core if it was present in 100% of the genomes at a match identity threshold of 95%.’ The genes were first aligned with mafft <https://pmc.ncbi.nlm.nih.gov/articles/PMC3603318/>.

6. Core SNP alignment – what was the number of SNPs in the final core SNP alignment?

The core gene alignment contained 267,355 variable sites (SNPs).

7. Mapping to reference genomes.
   a. References. Please check the details provided. FN432031.1 links to Salmonella enterica subsp. enterica serovar Typhimurium plasmid pSLT-BT, complete sequence. FN424405 links to Salmonella enterica subsp. enterica serovar Typhimurium str. D23580 complete genome as reported in Kingsley 2009 <http://www.genome.org/cgi/doi/10.1101/gr.091017.109>.

Thank you for pointing this mistake out. This has been changed in the text on line 253 to 254.

‘The reference genomes for *S*. Typhimurium ST313 was D23580 (Accession Number GCA 009953275 FN424405 [53]).’

b. How was mapping undertaken with BWA? Was it done using the GHRU pipeline (<https://www.protocols.io/view/ghru-genomic-surveillance-of-antimicrobial-resista-bp2l6b11kgqe/v3)>>?

We mapped sequencing reads to the ST313 reference genome (D23580, Accession Number GCA 009953275 FN424405 [47]) using BWA mem v0.7.17 (MapQ ≥ 20, excluding reads with secondary mappings). We performed indel realignment using GATK v3.7 IndelRealigner, de-duplication with Picard MarkDuplicates v1.126 (available at http://broadinstitute.github.io/picard/), and variant calling and consensus pseudosequence generation using samtools v1.6 and bcftools v1.6. We required a minimum of five supporting reads per strand and eight in total to call a variant - sites not meeting our filtering criteria were masked to ‘N’ in the final pseudosequence.

c. Given the complete reference genomes are available – unclear how the long read sequences were used in the mapping? Please explain. Or is it that the complete genomes for these two isolates were available at WSI?

Our apologies, this was an error. We used closed reference genomes for mapping, not long reads assemblies.

d. Any masking of phage or recombination in the ST313 and ST11 analyses?

Thank you for this question. There was no masking of phage or recombination of the ST313 and ST11 analyses.

e. What were used as outgroups for the trees and how were these treated in the analyses? Please provide details.

Thank you for the comment. The S. Typhimurium ST313 tree included in Supplementary Figure 1 was rooted to *S*. Typhimurium ST19 LT2. The S. Enteritidis ST11 tree was rooted to *S*. Gallinarum (Accession number SAMN08796416). The identity of the roots of these trees is included within the relevant figure legends.

8. How were the lineages in ST11 and ST313 identified? With contextual isolates that had previously been determined as having membership of a lineage? Please provide details of these contextual isolates and ensure the appropriate literature is cited.

Thank you for this comment. Yes, the lineages ST11 and ST313 were identified using the contextual isolates as detailed in the Supplementary Tables which have been attached to this resubmission. These tables contain a list of contextual genomes used, their lineage, and a reference to the literature in which they were originally published.

9. How were pairwise SNP distances calculated? Please ensure citation or acknowledgement of the tool (eg github if not published).

We used snp-dists (<https://github.com/tseemann/snp-dists>). This information has been included in the text in line 245.

Results

1. Important to highlight that looking at NTS only and that the Typhi isolates were not included. Is it correct that Typhi genomes were WGS but once confirmed as Typhi – were excluded from all downstream analyses?

For clarity, we have highlighted this at line 172 to 173 and at 275 to 277.

“As the focus of this study is NTS, whilst these isolates had the gene targets for *S*. Typhi, this data set will only discuss those isolates that yield NTS from sequencing.”

“In addition, one additional *S*. Typhi genome was identified and removed from the alignment, leaving a total of 270 NTS genomes for phylogenetic reconstruction.”

2. L212-213. Please revise this sentence for clarity as it initially read as if there were only three subspecies of Salmonella. The authors have this information at L219-220 – this could be moved up in this paragraph before getting into the details of serovars.

This has been changed to read as the following on lines 280 to 282:

“The collection of 270 genomes formed three phylogenetic clusters (Figure 2), corresponding to three of the five subspecies of *Salmonella enterica*. No isolates of *S*. *enterica* subsp. *arizonae*, *houtenae* or *indica* were identified. Neither were there any isolates of the other salmonellae species,  *S*. *bongori*.”

3. Was there any fluctuation in number of Salmonella samples detected in water sources by season over ~18 month timespan? Or not possible to tell from these data? The authors note the ST313 isolates were detected in 13/19 months of the study at L222.

It would be difficult to monitor this with the isolates studied as they are a subset of the total samples collected, and salmonella isolated from. For NTS as a whole, there were not enough positive samples to statistically determine seasonality.

4. L224 – please define MDR (3 or more drug classes)? Defined later at L256.

This has been addressed earlier in the text as requested, and the description can be found at line 293 “MDR, as defined by resistance to three or more antimicrobial classes”.

5. For the isolates where SISTR reported a mix of serovars, was and investigation undertaken to look at the surface antigen regions in the genomes? eg n = 3 Hissar | Choleraesuis | Paratyphi C | Typhisuis | Chiredzi | Rubislaw* and ST3695. Were the genome assemblies comprised of lots of contigs so could really tease apart, or was it possible to compare the regions to reference genomes to e.g. Choleraesuis and Paratyphi C. Could these isolates have a novel serovar? Especially as these three isolates are all staG in Table 4.

These three isolates were not *staG* positive genomically; we have changed how it was written on lines 367 to 371 and 384 to 385 for clarity.

“Sixteen of the 1,048 non-typhoidal *Salmonella* (1.4%) colonies isolated from 425/2,693 culture-positive samples were qPCR-positive for *staG.* All genomes were screened for the presence of *staG* *in silico.* The only serovar which contained the gene, *staG*, was *S*. Orion (seven genomes, Table 4, Figure 2). The remaining eight isolates that had positive amplification for *staG* by qPCR, lacked the gene when sequenced, as per Table 4.”

“Further isolates from different serovars were *staG* positive by qPCR *in vitro*, but unlike *S*. Orion, none of these contained *staG* in their genomes.”

6. L260-264 – discussion of lineages in ST313. These lineages and nomenclature 2.0 and 2.2 need to be explained to aid interpretation of results by readers unfamiliar with these lineages.

Thank you for this comment. Further explanation of the lineages and nomenclature with reference to relevant literature have been added to line 334-340:

“Since its first discovery, genomes of the sequence type ST313 has been further differentiated into lineages and sub-lineages. Chloramphenicol-sensitive ST313 lineage 1 was replaced in Malawi by chloramphenicol-resistant lineage 2 [59]. Subsequently, there has been emergence of ST313 sub-lineage 2.i within the Democratic Republic of Congo [6]. ST313 lineage 3, which is antibiotic-sensitive, emerged in 2016 [58]. Further phylogenetic analysis of lineage 2 isolates has documented the presence of the sublineages 2.2 and 2.3 which emerged between 2006-2008 and have been replacing lineage 2.0 [61].”

7. L281-286. Some of the information about staG should be in the Introduction (L283-284).

This description has been removed and superseded by an expanded section on the qPCR targets in lines 152 to 168.

8. Table 4 – what was the in silico analysis of staG and tviB positive genomes? Were the draft genomes screened for these two genes with blast? These details should be provided in the methods. L297 states in silico PCR was done on all assemblies? This correct? Were there any isolates that were positive for staG and tviB in silico that hadn’t been previously detected with PCR in vitro?

Our apologies, we used in_silico_pcr (available at <https://github.com/sanger-pathogens/sh16_scripts/blob/master/legacy/in_silico_pcr.py>) to screen all assembly contigs directly for predicted amplicons between the primer sequences. We have now added these details to the Methods section on lines 225 to 227.

“All assemblies were screened directly for predicted amplicons within the expected size range between the primer sequences in_silico_pcr (available at <https://github.com/sanger-pathogens/sh16_scripts/blob/master/legacy/in_silico_pcr.py>) was used.”

There was one isolate of *S*. Orion ST639 which was genomically positive for *staG* but negative for *staG* on *in vitro* PCR (see Table 4, page 20).

9. Figure 1 had a lot of information in it. Would suggest breaking this into 2 figures.
   a. The tree with the tips coloured by subspecies, serovar and ST
   b. AMR – details of those with any AMR mechanism detected. From the AMR table – there are 5 serovars with AMR and 1 S. salamae isolate with any AMR. Potentially show as barplots facetted by serovar where any AMR was detected. This would help to highlight that S. Isangi had lots of AMR mechanisms detected include blaCTX-M-14
   c. Why was ttr data not included when staG and tviB presence / absence from PCR included? This would also be nice to see visualised.

Thank you very much for this comment, these are very interesting suggestions.

To improve the clarity of this figure, we have now added Serotype- and ST- specific clade labels to the major groups. The suggestion to make barplots is interesting, but since we have many different gene and ST groupings, this would quickly become unreadable. Instead, we now provide a new Figure 3 that shows both sample size and proportion with resistance markers for each ST.

We have opted to maintain the tree, now Figure 2, as a single entity rather than dividing into two to maintain all of the information within a single location, which clearly outlines the specific serovars and sequence types which carry genomic AMR determinants.

We feel that the presence of *S*. Isangi carrying genomic determinants of AMR resistance is highlighted clearly in Figure 1. In order to improve the clarity of the tree we have included *Salmonella* subspecies on the tip labels and maintained serovar and ST within the heatmaps.

*ttr* data was not included as, as expected, all genomes were confirmed to be *ttr* positive (Table 1, page 9). Therefore, given the complexity of the graph, it was felt that this information did not contribute any additional information than that within the rest of the paper.

10. Supplementary Fig 1-2. The authors have integrated previously published data used to infer lineage and epidemic clade. Details of the inferred lineage / clade membership should be reported for the isolates in a supp table.

Thank you for this comment. The genomic data used to inform the lineages of *S*. Enteritidis ST11 and *S*. Typhimurium ST313 were identified using the contextual isolates as detailed in the Supplementary Tables which have been attached to this resubmission. These tables contain a list of contextual genomes used, their lineage, and a reference to the literature in which they were originally published.

11. Supplementary Table 2 referred to at L211. Unsure if this is the total genomes IDs table?

This was a version error and has been removed.

12. Would be useful to include a column that highlights QC pass – assume the NA in columns from Genome.Name onwards are the ones that failed?

For clarity, NA has been changed to *Did not pass QC*

13. Which genomes were ‘duplicate’ genomes and removed? Could these be noted in this table as well.

This suggestion has been included in the now combined Supplementary Table 1 containing all isolate information and associated metadata.

14. AMR data (supp table 3?) only has 59 rows. From Fig 1 – appears the other genomes were susceptible. However, it is important useful to include and report these data for all isolates. Likewise, the ttr, staG and tviB not reported for all and should be included in this table (or Supp table 2).

AMR Data and Isolate Data have been combined into one Supplementary Table 1, which includes absence as well as presence of AMR associated genes and SNPs and *in silico* presence or absence of qPCR primer targets.

Conclusions

1. The conclusions are largely supported by the results. Additional clarification of methods and results will help. The public health relevance was clearly addressed.
2. New concepts introduced in the discussion – group C and group F. Should be backgrounded in the introduction if discussing.

These were not relevant to the paper, and we have therefore decided to remove the discussion of Group C and F serovars due to its expanding beyond original scope.

3. Limitations of the study were stated. The impact of the sequencing strategy could be explored a little more at L355.
4. The strengths of this study could also be highlighted in the limitations paragraph.

We have attempted to expand on limitations and strengths as requested.

Editorial and Data Presentation Modifications

1. Title – there are two titles on the first page (L1-L2). Think the first is the full title and the second is the short running title? If not – please address.

This is correct, and was done for the submission, these have been clarified further.

2. Abstract. Suggest some edits to make more accessible to PLoS NTD readers.
3. Explaining terms in abstract. Switches from Typhi (L29) to NTS (L31) without making clear the difference. Uses iNTS (L39) without explaining what iNTS is and how it relates to Typhi and NTS. The full introduction explains it nicely

Whilst important to the story, for the sake of clarity in the Abstract, mentions of Typhi have been removed, to instead focus on the non-Typhoidal Serovars this paper discusses exclusively.

4. L27-28 reads more suited to the discussion as this manuscript explored the ability for environmental samples to inform genomic surveillance

This has been moved as suggested.

5. The results part of the abstract could be revised. Unclear what the contemporaneous surveillance was – should this be mentioned in methods? And L41-L42 focus on lineages within ST313 which is S. Typhimurium and iNTS.

This has also been removed for clarity.

6. L85 – Typhi and NTS serovars linked to BSI. What about Paratyphi A Band C?

Term has been changed to typhoidal *Salmonella*, and Paratyphi has been included under that terminology: “Where surveillance has been established, both Typhoidal *Salmonella* (inclusive of *S*. Typhi and *S*. Paratyphi A, B and C) and NTS serovars have often been identified as common causes of bloodstream infection” in Line 82 to 83.

Summary and General Comments

1. Ethics. No statement was included. Do not think required as environmental surveillance of Salmonella from water sources.

The details of an ethics waiver has been included in lines 112 to 114.

2. Data availability. The authors have made a good effort at making the data available in this study. In the results section additional details were requested. Briefly these are:
   a. Accessions provided in AMR table (details of 59 isolates). Total genome tables has details for 341 isolates but accessions for each not provided. There look to be thousands of isolates in PRJEB37378 (the associated bioproject).
   b. Please ensure each of the 339 genomes in the study have full information for AMR profiles (inferred R/ S from genotype) and presence/ absence of PCR genes (ttr, tviB and staG) in the supplementary tables.
   c. Details of ‘reads published elsewhere’ reported at L154 that were used for context

The genomic data used to inform the lineages of *S*. Enteritidis ST11 and *S*. Typhimurium ST313 were identified using the contextual isolates as detailed in the Supplementary Tables which have been attached to this resubmission. These tables contain a list of contextual genomes used, their lineage, and a reference to the literature in which they were originally published.

Thank you for your feedback and consideration.

Kind Regards

Jonathan Rigby
